# Beyond Work: The Role of “Family-Friendly” Practices in the Subjective Well-Being of Teleworkers and On-Site Workers in the COVID-19 Pandemic

**DOI:** 10.3390/ijerph21040447

**Published:** 2024-04-05

**Authors:** Sílvia Lopes, Rita Couto, Anabela Rodrigues, Ana Sabino, Íris M. Oliveira, Paulo C. Dias, Ângela Leite, Vânia Sofia Carvalho

**Affiliations:** 1 CICPSI, Faculdade de Psicologia, Universidade de Lisboa, Alameda da Universidade, 1649-013 Lisboa, Portugal; ritacouto@edu.ulisboa.pt; 2 CEFH—Centro de Estudos Filosóficos e Humanísticos, Faculdade de Filosofia e Ciências Sociais, Universidade Católica Portuguesa—Centro Regional de Braga, 4710-302 Braga, Portugal; anabela.rodrigues@ucp.pt (A.R.); imoliveira@ucp.pt (Í.M.O.); pcdias@ucp.pt (P.C.D.); aleite@ucp.pt (Â.L.); 3 School of Psychology, ISPA Instituto Universitário, R. Jardim do Tabaco 34, 1149-041 Lisbon, Portugal; asabino@ispa.pt; 4 APPsyCI—Applied Psychology Research Center Capabilities and Inclusion, ISPA Instituto Universitário, 1149-041 Lisboa, Portugal

**Keywords:** teleworkers, “family-friendly” practices, organisational support, supervisor support, subjective well-being, work engagement, COVID-19

## Abstract

During the COVID-19 pandemic, telework emerged as a pivotal strategy to mitigate the spread of the virus. However, telework’s feasibility was contingent on job roles. This gave rise to two distinct groups: teleworkers and on-site workers. However, the impacts of social support and well-being extended to both groups. This study investigated the link between organisational and supervisory family support and subjective well-being, examining work engagement as a mediator. Conducted in Portugal, this cross-sectional study surveyed 515 individuals via web-based questionnaires. Data were analysed using descriptive statistics, correlation analysis, confirmatory factor analysis, and multiple-group analysis. The findings revealed a positive correlation between perceived organisational family support (POFS) and work engagement for both groups. Additionally, perceived supervisory family support (PSFS) positively correlated with work engagement for telecommuters but not on-site workers. Furthermore, work engagement was positively associated with subjective well-being for both groups. Moreover, work engagement mediated the relationship between POFS and subjective well-being. This study enriches the literature by analysing POFS, PSFS, work engagement, and subjective well-being dynamics among teleworkers and on-site employees.

## 1. Introduction

During the emergence of the COVID-19 pandemic, there was a clear need to adopt telework, either fully or partially. This aimed to reduce the spread of the virus and its effects [1,2,3,4]. Telework was deemed feasible when the employee’s circumstances permitted it and where their roles were suitable for remote employment [5]. Independently and autonomously, telework allows workers to carry out their professional activities away from the organisation’s premises, facilitated through information and communication technologies (ICT) [6]. In this context, telework was implemented abruptly and unfamiliarly, impacting people’s lives, particularly in terms of family dynamics, as, for many, this was their first encounter with telework. The main challenges included a lack of interaction with colleagues, time management, and balancing work with family life [7,8,9].

On the other hand, not all jobs allowed for the adoption of telework, requiring the performance of duties on-site. In other instances, telework was only resorted to during quarantine periods, with a return to in-person work when feasible. Confronted with a scenario wherein quarantine periods could become recurrent, individuals found their personal, familial, and professional spheres subject to external and unpredictable forces stemming from the pandemic. These effects impacted teleworkers and those who worked in a traditional in-person setting.

Considering the prevailing circumstances, the interplay between work and family dynamics is marked by the gradual dissolution of boundaries, requiring individuals to achieve an equilibrium between these spheres. The relevance of effectively managing this relationship becomes apparent when it is recognised that family, work, and their reciprocal influence constitute critical determinants of satisfaction and well-being [10,11]. Consequently, to address the challenges inherent in this interconnection, numerous organisations have implemented measures designed to facilitate this interplay. These measures, indicative of a family-supportive culture, are manifested as family-friendly practices, implemented diversely, thereby conferring manifold benefits upon employees [12,13].

Within this context, the supervisor’s role in this domain will likely contribute to the perception that an organisation is aligned with familial issues. The affirmative actions undertaken by supervisors play a crucial role in mitigating the work–family conflict (WFC) experienced by a substantial cohort of workers, consequently fostering an improvement in their overall well-being [14]. Hence, any assistance provided or perceived by employees to address their familial responsibilities may originate from two sources: the organisation, referred to as perceived organisational family support (POFS), and the supervisor, identified as perceived supervisory family support (PSFS).

Additionally, the COVID-19 pandemic provides a unique context through which to explore the impacts on well-being, serving as a warning to ensure the safeguarding of this variable during a crisis, especially for those engaged in telework. It can also yield valuable insights for employers to enhance the well-being of their employees [15,16]. In the present study, we conceptualise subjective well-being as a cognitive self-assessment of overall life across different domains, such as life achievements, personal relationships, work, and health [17]. Simultaneously, human resource managers have prioritised the engagement of their employees, specifically in the work domain [18]. Work engagement is characterised as a psychological state of fulfilment and a favourable disposition towards one’s tasks at work, encompassing high levels of physical, cognitive, and emotional energy [19,20,21].

Moreover, in a pandemic context that has caused radical changes in family life, it becomes imperative to safeguard both subjective well-being and employee engagement. The unpredictability of temporal events generates uncertainties in both the personal and professional domains [9,22]. Given that tranquillity in the family is essential in preserving the work domain and vice versa, it is crucial for organisations, specifically supervisors, to exhibit supportive behaviours towards families. Beyond being recommended in organisational contexts [14], such behaviours profoundly influence the variables above, thereby enhancing or diminishing individuals’ perceptions of support. Furthermore, in such an atypical context, where resilience proves to be crucial, the support of supervisors is highlighted as they can foster such resilience [23,24].

Following the theoretical framework of the Job Demands Resources (JD-R) theory proposed by Bakker and Demerouti [25], the primary objective of this study is to clarify the association between perceived organisational family support (POFS) and perceived supervisory family support (PSFS) and subjective well-being. Furthermore, the study seeks to empirically examine the mediating role of work engagement within the relationships mentioned above, while studying the moderating role of distinct work modalities, distinguishing between in-person and telework arrangements.

### 1.1. The “Family-Friendly” Organisational Culture: The Impact of the COVID-19 Pandemic

According to Greenhaus and Beutell [26], the concept of work–family conflict (WFC) can be seen as a clash between roles, wherein pressures arising from work and family can be incompatible, stemming from issues related to time, tension, and/or behaviour. In response to the consequences of WFC, organisations may adopt measures to address the challenges in the work–family relationship.

These measures include leave, work flexibility, and conventional practices, collectively called “family-friendly practices/policies” [13]. However, beyond adopting these measures, organisations must have the necessary conditions for their applicability, which implies fostering a family-supportive culture [27,28,29]. In other words, when family life issues are acknowledged, organisations value them, providing proper support for challenges in this sphere while also doing the same in the work sphere, shaping employees’ beliefs and values regarding these aspects [29]. Family-friendly practices must be conceptualised as an integral part of the family-supportive culture, characterised by its formal norms. On the other hand, the informal norms of this culture encompass all existing organisational norms that need to be formally established—for instance, support from managers or supervisors [12,27,30,31].

From an organisational perspective, the implementation of these practices is crucial as it makes companies more attractive by demonstrating concern for their employees, thereby stimulating favourable behaviours and attitudes with significant benefits for both sides [27,29,31,32]. 

However, family-friendly practices have acquired a new context of operation due to the COVID-19 pandemic. Since the pandemic has accentuated the ambiguity of the work–family relationship, it implies that these practices must be reconsidered and evaluated to address the challenges of the new context involving the workplace, which can vary depending on the type of work arrangement—whether it be in-person or telework.

During the pandemic, individuals who had to perform their duties in person dealt with different sources of stress: on the one hand, there was a higher risk of infection due to increased exposure to potentially unwell individuals [33,34,35], elevating the risk of transmitting the virus to their family members at home. On the other hand, since they had to commute to work, their contact with their families was reduced during this period, leading to increased concern and stress as they relied on the presence of family members at home and could not assist them when needed. For instance, parents working in person may have struggled to supervise their children engaged in remote schooling.

For these reasons, Avdiu and Nayyar [36] considered working in person during the pandemic a psychosocial risk due to the fear of contagion and the new social interaction conditions. On the other hand, considering perspectives prior to the pandemic, studies have shown that the presence of children during telework leads to increased pressure, lower satisfaction among couples, and, specifically for females, an increase in household and/or family responsibilities [37,38]. Facing a reality that requires other family members at home, there are more interruptions and distractions, increasing the levels of work–family conflict for many teleworkers [3,16].

Considering the above, the COVID-19 pandemic has exposed individuals to stress and uncertainties, where social support plays a crucial role in their resilience [39]. Hence, as previous perspectives have suggested, participative and supportive management, encouraging employee engagement, is sufficient for the emergence of flexible and life-enhancing work arrangements in the current context, where two prevalent types of work arrangements (i.e., in-person and telework) co-exist within the same organisation [9,40]. 

Some authors argue that organisations lacking a work–family culture have negative impacts on their employees, such as emotional fatigue, leading to stress and impacting their well-being, ultimately affecting their job performance [41,42,43,44,45,46]. 

### 1.2. Job Demands Resources Model 

How individuals engage with work and family dynamics influences their work well-being, which inevitably reflects their overall well-being beyond work [12]. One theory that explains well-being at work and beyond it is the Job Demands Resources (JD-R) theory [25,47,48]. The theory assumes the principle that the elements of work can be perceived as resources or demands that influence the well-being of individuals. It encompasses two processes: the health impairment process and the motivational process.

The health impairment process explains the emergence of burnout, as work demands wear down workers’ physical and mental health. The health impairment process also allows us to understand that work–family conflict (WFC), characterised as the adverse effect of the work domain on the family domain, is related to burnout and lower life satisfaction [12,20,49,50,51,52]. On the other hand, the motivational process, explained by the presence and perception of work resources, such as social, emotional, and instrumental support, induces work engagement [53]. These resources are crucial in reducing work demands by stimulating a positive emotional state, thereby promoting work engagement [25].

Social support is essential in stimulating work resources, which this study assumes as perceived organisational family support (POFS) and supervisory family support (PSFS). This relationship can be explained by the fact that family-supportive work environments positively influence general well-being through mediating work engagement [54].

Additionally, as Demerouti and Bakker [55] noted, in a crisis such as the COVID-19 pandemic, job characteristics in isolation are inadequate in elucidating employee health and motivation, which are the primary focal points of the Job Demands Resources (JD-R) theory. Instead, the interplay between demands and resources at the individual, familial, occupational, and organisational levels predict outcomes [55]. Furthermore, alongside individual regulatory strategies, the regulatory strategies of the family, leadership, and organisation or team are posited to mitigate the effects of demands and resources on outcomes [55]. Thus, in a crisis, we may assume that organisational and supervisory family support is even more crucial in explaining individual outcomes such as work engagement and subjective well-being.

### 1.3. Perceived Organisational Family Support and Work Engagement

An organisational climate supportive of family emerges as a pertinent subject, especially as it plays a more important role in reducing work–family conflict (WFC) than general organisational support [56]. Perceived organisational family support (POFS) can be defined as employees’ perceptions of the assistance provided by their respective organisations to their families, encompassing various forms of support, namely instrumental, informational, and emotional [57].

Family-supportive organisations encourage employees’ engagement in work and family responsibilities, diminishing potential tensions between the two roles and reducing WFC [26]. Family-supportive organisations are particularly important for employees who emphasise work tasks more, avoiding a lack of harmony between their career and family expectations [26,54]. 

Generally, workplaces lacking support for employees lead to many adverse outcomes that threaten the work–family equilibrium [58]. Specifically, in the context of work–family conflict, there exists an impact on an employee’s involvement in family life. The threat to the work–family equilibrium is discernible in terms of reduced time dedicated to family activities, stemming from heightened demands within the work domain. Such constraints are exacerbated by heavy workloads and performance pressures, resulting in increased stress levels. The heightened stress impairs individuals’ emotional capacity to engage in activities outside work, especially those related to family life [26,58,59]. This scenario transcends differences in work modalities, as the dominance of work-related demands over familial responsibilities persists regardless of the work setting. Remarkably, the absence of organisational support in this facet exacerbates the emergence of work–family conflict (WFC). Noteworthy findings by certain scholars indicate that parents perceiving the lack of a family-supportive culture tend to extend their working hours compared to those who perceive such support [57].

When individuals experience WFC, they are likelier to present diminished energy levels for work investment, i.e., work engagement [54,60,61]. Work engagement is a positive psychological state comprising elements invested in work to achieve goals, such as high energy levels, persistence, and identification [25].

Several studies demonstrate that POFS mitigates challenges associated with balancing work and family responsibilities [54,62,63,64]. Therefore, we posit that POFS constitutes a resource that stimulates the acquisition of other resources, providing flexibility for professionals to handle the demands of both work and family. Due to the motivational process in the JD-R theory, this support provided by organisations fosters the development of work engagement. This argument is in line with the Conservation of Resources theory (COR; Hobfoll, [65,66]), which postulates a gain spiral in the acquisition of resources. Indeed, the Job Demands Resources (JD-R) theory aligns closely with the Conservation of Resources (COR) theory. More precisely, as Xanthopoulou et al. [67] noted, if the principle of the Conservation of Resources (COR) theory is incorporated into the motivational mechanism of the JD-R model, it could be predicted that the presence of job resources would foster the accumulation of resources, thereby leading to more positive outcomes.

Hence, we present the following hypothesis.

**H1.**  *Perceived organisational family support has a positive relationship with work engagement for employees in telework (H1a) and on-site (H1b)*.

### 1.4. Perceived Supervisory Family Support and Work Engagement

The COVID-19 pandemic has impacted human life in all aspects. From the familiar sphere perspective, although remote work can be considered a practice aimed at balancing the work–life relationship, teleworkers may easily feel that work intertwines with their family dynamics and vice versa, hindering balance in the aforementioned relationship [68,69,70]. Indeed, a study carried out by Edvardsson and Gardarsdottir [71] demonstrated that leaders’ attitudes towards telework, along with other factors such as adequate equipment and training, were significant determinants of positive outcomes regarding the telework experience during the COVID-19 pandemic. On the other hand, due to the insecurity, stress, and general concern provoked by the pandemic, even those who worked on-site were likely to experience the erosion of work–family boundaries. In this regard, since the support provided to employees shapes the amount of stress at work, relationships with supervisors play a crucial role in reducing work demands, as they also influence work attitudes and commitment [72,73,74]. Although it is recognised that interpersonal relationships profoundly impact organisational behaviour [35], many professionals may have encountered challenges in fulfilling their responsibilities upon transitioning to a new work context during the COVID-19 pandemic. These difficulties arose irrespective of whether employees worked remotely or on-site [74].

The relevance of the theme of leadership becomes more prominent when contextualised within the pandemic, since leadership has affected interpersonal relationships within and outside the organisation [75]. In other words, a leader exerts an influence on an individual’s life beyond work, which can contribute to reducing work–family conflict (WFC) and/or enhancing work–family enrichment (WFE) [12]. Thus, it is emphasised that the sensitivity and awareness of supervisors regarding this topic contributes to the harmony of the work–family relationship by embodying the so-called family-supportive supervisor behaviours (FSSB), which encompass all behaviours adopted by supervisors, affording support to the families of their subordinates [76,77]. These behaviours comprise four dimensions: emotional, instrumental, role modelling, and creatively managing the work–family relationship [76,77]. Emotional support revolves around fostering secure communication and understanding regarding employees’ work–life balance needs. Instrumental support involves supervisors responsively addressing their employees’ work–life balance needs in a transactional manner. Role modelling entails supervisors exemplifying the effective integration of work and personal life. Creative work–family management involves implementing proactive, individualised strategies to assist employees in balancing their work and personal responsibilities [76,77].

Therefore, when they perceive that their supervisors genuinely care about and value the needs of their families, exhibiting supportive behaviours towards them, employees are encouraged to integrate, rather than separate, work and family life. Consequently, they can leverage the resources provided by both domains [78], so that their performance in multiple life roles is advantageous [12]. This clarifies the existence of a positive association between family-supportive supervisor behaviours (FSSB) and work–family enrichment (WFE) [78,79], which can be explained by the paradigm itself, as experiences in one role enhance the quality of life in the other [26]. This is further elucidated by role accumulation theory [80] and the expansionist hypothesis [81,82]. Examples of FSSB in the context of in-person work include taking calls about family matters, while, in teleworking situations, it could involve the option to suspend teleworking hours to accommodate children’s remote learning needs.

Once family-supportive supervisor behaviours (FSSB) are established, the conditions are conducive to engagement, as evidenced by Straub’s foundations [79], where engagement is a consequence of FSSB. In addition, Matthews et al. [83] found a direct relationship between FSSB and engagement. This relationship can be explained by the fact that FSSB stimulates employees to protect and acquire new resources to effectively manage the work–family duality and improve their quality of life [78]. Promoting this climate encourages professionals to explore new ways of thinking and obtaining resources and fosters the perception of work engagement [83,84]. Similarly, Timms et al. [85] also concluded that when employees experience a positive work–family relationship, they report higher levels of work engagement.

According to the Job Demands Resources (JD-R) perspective, the presence of family-supportive supervisor behaviours (FSSB) constitutes a vital resource through the motivational process. It allows professionals to experience fewer difficulties in managing their work and family responsibilities, activating professional motivation through engagement. Consequently, due to experiencing fewer challenges in coordinating their responsibilities, they feel greater satisfaction with the work–family balance [54,86]. 

Thus, we posit the following hypothesis.

**H2.**  *Perceived supervisory family support has a positive relationship with work engagement for employees in telework (H2a) and on-site (H2b)*.

### 1.5. Work Engagement and Subjective Well-Being

High levels of energy, persistence, identification, and goal-directed motivation are some elements related to work that constitute a positive emotional state, defining work engagement [25]. Additionally, according to Schaufeli et al. [87], work engagement can be characterised by vigour (referring to high levels of energy and resilience during work, even in the face of difficulties), dedication (experiencing a sense of enthusiasm, pride, and challenge due to being involved with work), and absorption (being so deeply engaged and focused on work that time passes quickly, and individuals may even experience difficulties in separating from work).

Similarly, every individual values their well-being throughout life and seeks to place it at the core of their needs and motivations [88]. This is because well-being influences various aspects of life, including better health and a longer life expectancy, more robust social connections, active social participation, and improved work performance [89]. Subjective well-being is defined by an individual’s evaluation of their overall life satisfaction, which encompasses both affective and cognitive components [89]. Life satisfaction, which represents the cognitive component, is widely acknowledged and empirically validated as a crucial indicator of subjective well-being [90]. In this context, several measures of subjective well-being have been created. The Personal Well-being Index (PWI) is among the most employed measures [91]. The PWI assesses individuals’ life satisfaction across different domains: standard of living, health, life achievements, personal relationships, personal safety, community, future security, and religion/spirituality [17]. These domains encompass individuals’ overall life satisfaction [91].

The rapid spread of the COVID-19 pandemic has caused a global crisis with long-term effects. Individuals have been affected by this situation, experiencing unexpected challenges in terms of physical and psychological health, as well as well-being [92,93,94,95,96]. This aspect aligns with previous COVID-19 pandemic research, indicating a positive relationship between work engagement and positive mental and physical states [19]. In addition, during the COVID-19 pandemic, research conducted by Chidambaram et al. [97] with a sample of employees in the information technology (IT) sector revealed a negative relationship between pandemic-induced stress and work engagement and a positive relationship between telework and work engagement. Likewise, Chambel et al. [98] utilised a sample comprising 435 bank employees who were surveyed before the onset of the pandemic and again approximately ten months thereafter. Their findings indicated that the transition to telecommuting positively impacted work engagement among employees who shifted from on-site work to telework, in contrast to those who continued to work on-site. Furthermore, Wang et al. [99] suggested that individuals who experienced increased workloads due to remote work reported lower levels of well-being. Additionally, Hadi et al. [100] noted that the daily job and home demands during telework are positively correlated with emotional exhaustion (i.e., lower levels of well-being). Despite the distinct conceptualisations of both concepts (i.e., work engagement and well-being), they share a common aspect: in their presence, professionals exhibit a positive state that beneficially influences outcomes both at the workplace and beyond [101].

In summary, work engagement is associated with personal resources in the workplace, which create a positive emotional state that contributes to the overall level of individuals’ well-being [102,103]. 

This leads us to formulate the following hypothesis.

**H3.**  *Work engagement positively correlates with subjective well-being for employees in telework (H3a) and on-site (H3b)*.

### 1.6. The Mediating Role of Work Engagement

Work resources have consistently been positively associated with work engagement [104]. In the present research, these resources are assumed in the form of perceived organisational family support (POFS) and perceived supervisor family support (PSFS), consistent with other authors, who have noted that social support is a vital work resource [20]. Previous studies have shown that work resources, mainly the support offered and perceived by employees, serve as intrinsic motivation for the individual and facilitate employees’ engagement in their work, mitigating the impact of job demands on physical and psychological health [83,105]. Additionally, there is evidence that those who perceive supervisor support experience positive emotions [106,107], which can be extended to encompass the concept of subjective well-being, also measured in this research. Similarly, meta-analytic investigations demonstrate that work engagement is strongly associated with increased employee well-being and work-related behaviours [108,109].

Therefore, POFS and PSFS are expected to relate to individuals’ subjective well-being regardless of the modality in which employees work (i.e., telework, and on-site). In addition, due to the support provided by the organisation (i.e., POFS) and the supervisor (i.e., PSFS), individuals can allocate time and energy to deal with work demands and present higher work engagement. In turn, work engagement will be translated into higher levels of subjective well-being. 

Thus, we posit the following hypotheses.

**H4.**  *Work engagement mediates the relationship between POFS and subjective well-being for employees in telework (H4a) and on-site (H4b)*.

**H5.**  *Work engagement mediates the relationship between PSFS and subjective well-being for employees in telework (H5a) and on-site (H5b)*.

## 2. Materials and Methods

### 2.1. Data Collection and Participants

Data were collected through an online questionnaire on the Qualtrics platform in May 2020. The research sample was obtained by using a convenience sampling approach and, thus, non-probabilistic sampling. We presented this study to twelve companies operating in several activity sectors in Portugal, including the industrial and services sector and public and private companies. After accepting and entering this study, the companies received an e-mail containing the link to the online survey, and the companies forwarded this e-mail to their employees. Ethical aspects were taken into consideration for the development of this study. The confidentiality of the answers was ensured since only the research team had direct access to the data. Moreover, no personal information that would compromise the anonymity of the answers was collected (e.g., participants’ and company names). There was no incentive (cash or otherwise) for participation in this project.

We obtained 515 answers, namely 411 answers from employees in telework and 104 answers from employees in on-site work. The sample was composed mainly of women (74.6%). The participants were, on average, approximately 40 years old (SD = 9.45) and, for the most part, possessed a bachelor’s degree (48.5%) or a higher level of education completed (29.3%). Most participants were married/in a partnership (70.6%) and had children (62.7%). Additionally, most individuals had a permanent contract (74.0%) and a job tenure of more than ten years (36.9%). A description of the sample in total and across groups is reported in Table 1.

### 2.2. Instruments

#### 2.2.1. Perceived Organisational Family Support

To assess perceived organisational family support (POFS), we used 6 items from the instrument developed by Jahn et al. [57], which was adapted to Portuguese for the present study. Three items reflected the instrumental and informational support of the organisation (e.g., “My organisation has many programs and policies designed to help employees balance work and family life”). The remaining three items reflected the emotional support of the organisation (e.g., “In general, my organisation is very supportive of its employees with families”). The response format was a 7-point scale (1 = strongly disagree and 7 = strongly agree). The scale presented excellent reliability (α = 0.94). 

#### 2.2.2. Perceived Supervisory Family Support

To assess perceived supervisory family support (PSFS), we used 5 items from the instrument developed by Jahn et al. [57], which was adapted to Portuguese for the present study. An example item is “My supervisor is very understanding if someone has to leave early or come in late due to a family emergency”. The response format was a 7-point scale (1 = strongly disagree and 7 = strongly agree). The scale presented excellent reliability (α = 0.90).

#### 2.2.3. Work Engagement

To assess work engagement, we used 3 items from the ultra-short instrument developed by Schaufeli et al. [110]. We used the Portuguese-validated version used by Lopes et al. [111]. An example item is “At my work, I feel a burst of energy”. The response format was a 7-point scale (1 = never and 7 = always, every day). The scale presented satisfactory reliability (α = 0.77).

#### 2.2.4. Subjective Well-Being

To assess subjective well-being, we used the Personal Well-being Index (PWI; International Well-Being Group [91]) and the single-item scale of Overall Life Satisfaction (OLS; International Well-Being Group [91]). We used the Portuguese-validated version used by Bedin and Sarriera [112]. The Personal Well-being Index measures subjective well-being within different satisfaction domains, namely standard of living, health, life achievements, personal relationships, personal safety, community, future security, and religion/spirituality. Each of the eight domains was assessed by one item, resulting in a scale of 8 items. An example item is “How satisfied are you with your standard of living?”. The response format was a 10-point scale (1 = no satisfaction at all and 10 = completely satisfied). The scale presented good reliability (α = 0.86). The single-item scale on Overall Life Satisfaction consisted of a question in the following terms: “Thinking about your own life and personal circumstances, how satisfied are you with your life as a whole?” The response format was also a 10-point scale (1 = no satisfaction at all and 10 = completely satisfied). 

#### 2.2.5. Control Variables

We controlled the following variables: “sex” (0 = men; 1 = women), “age” (measured in years), “educational level” (from 1 = 9th grade to 5 = doctoral degree) “single” (1 = yes; 0 = no), “children” (1 = yes; 0 = no), and job tenure (from 1 = under 1 year to 5 = over 10 years).

## 3. Results

### 3.1. Descriptive Statistics and Correlations

Table 2 presents the means and standard deviations and the test to compare the groups. As is possible to observe, significant differences were found between the two groups; namely, teleworkers showed a higher level of POFS and PSFS compared to on-site workers (t = 4.39, *p* < 0.01; t = 4.33, *p* < 0.01; respectively). No significant differences were found between the two groups concerning the other studied variables. 

Regarding the correlation matrix (Table 3), the correlations were generally consistent with the theorised pattern of relationships, and the correlations among the variables also demonstrated no multicollinearity problems (r < 0.80; [113]). Additionally, we calculated the variance inflation factor (VIF) for each studied variable, and the values obtained varied from 1.18 to 1.65 and were, thus, smaller than 10 [114], suggesting no multicollinearity issues.

The control variables contributed significantly towards explaining the variance. Concerning the teleworker group, we found a negative and significant relationship between sex and POFS (r = −0.13, *p* < 0.05) and sex and work engagement (r = −0.12, *p* < 0.05). These results seem to indicate that men presented higher POFS and work engagement when compared to women, in the teleworker group. In addition, age showed a positive and significant relationship with work engagement (r = 0.17, *p* < 0.01) and a negative and significant relationship with PWI (r = −0.11, *p* < 0.05). As such, older teleworkers may present higher work engagement but lower PWI compared to younger teleworkers. Furthermore, the results seem to indicate that the higher the educational level of teleworkers, the lower their POFS (r = −0.10, *p* < 0.05). Moreover, having children also seems to contribute to a decrease in teleworkers’ PWI (r = −0.13, *p* < 0.05). However, the higher the teleworkers’ job tenure, the higher the work engagement that they present (r = 0.11, *p* < 0.05). Finally, concerning the on-site worker sample, a positive and significant relationship was observed between age and work engagement (r = 0.29, *p* < 0.01). As such, in line with what was observed in the teleworker sample, older on-site workers may present higher work engagement compared to younger on-site workers.

### 3.2. Confirmatory Factor Analysis

Confirmatory factor analysis (CFA) was performed using the AMOS 26 software to evaluate the convergent and discriminant validity of the measurement model. The measurement model obtained a good model fit [X2 (214) = 828.91, *p* < 0.01; SRMR = 0.05; IFI = 0.93; CFI = 0.93; RMSEA = 0.08]. Then, the measurement model was confronted with an alternative one-factor model (with all items of each studied variable loading into one latent factor). This alternative model exhibited a poor fit to the data [χ2 (223) = 3493.42, *p* < 0.01; SRMR = 0.17; IFI = 0.63; CFI = 0.63; RMSEA = 0.17]. As such, the research variables’ factor structures aligned with the conceptual model since the manifest variables loaded, as intended, on the latent variables.

### 3.3. Test of Hypotheses

**Figure 1:**
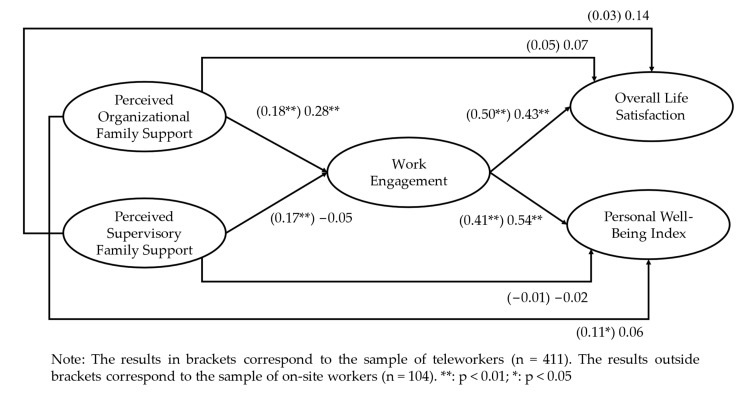
Unstandardised estimates for the multigroup model.

We performed a multiple-group analysis using AMOS (version 26.0) to test the hypotheses. The instructions of Byrne [115], already used in previous studies [116,117], were followed. The multigroup structural model, which included the two groups analysed [χ2 (428) = 1090,913, *p* < 0.01; SRMR = 0.06; IFI = 0.93; CFI = 0.93; RMSEA = 0.06], showed a good fit to the data. All the structural paths in the unstandardised coefficients are presented in Figure 1.

H1 predicted that POFS would have a positive relationship with work engagement for employees in telework (H1a) and on-site (H1b). The result was in line with the prediction (B = 0.18, *p* < 0.01; B = 0.28, *p* < 0.01; respectively) and lent support to H1a and H1b (Figure 1). However, concerning the relationship between PSFS and work engagement (i.e., H2), a positive and significant relationship was only observed in the teleworker group (B = 0.17, *p* < 0.01), while this relationship was not significant for the on-site worker group (B = −0.05, n.s.). Thus, H2a was supported and H2b did not receive support from the data.

Regarding H3, this hypothesis predicted that work engagement would have a positive relationship with subjective well-being both for employees in telework (H3a) and employees on-site (H3b). As previously noted, subjective well-being was measured using two variables, namely overall life satisfaction and personal well-being. Regardless of the indicator of subjective well-being used, as is possible to observe in Figure 1, a positive and significant relationship was obtained between work engagement and overall life satisfaction and between work engagement and personal well-being, for both the teleworker group (B = 0.50, *p* < 0.01; B = 0.41, *p* < 0.01, respectively) and the on-site worker group (B = 0.43, *p* < 0.01; B = 0.54, *p* < 0.01, respectively). Thus, H3a and H3b were supported.

In Table 4, it is possible to observe the indirect effects of the mediation models tested. Concerning H4, all indirect effects were found to be significant since the 95% confidence interval of the indirect effects did not include zero. As such, work engagement is a variable that contributes to explaining the relationship between POFS and subjective well-being for the two studied groups (i.e., teleworkers and on-site workers). Hence, H4a and H4b were supported. However, the same pattern of results was not observed regarding the hypothesised mediating role of work engagement in explaining the relationship between PSFS and subjective well-being (H5). More precisely, the indirect effect PSFS → Work Engagement → Subjective Well-Being [PWI measure] (effect size = 0.07, CI [0.02; 0.14]; Table 4), as well as the indirect effect of PSFS → Work Engagement → Subjective Well-Being [OLS measure] (effect size = 0.09, CI [0.03; 0.16]; Table 4), was only significant for the teleworker group. This result indicates that, for on-site workers, only POFS and work engagement seem to directly contribute to increasing these individuals’ subjective well-being (see Figure 1). As such, H5a was supported and H5b did not receive support from the data.

## 4. Discussion

This study examined the relationship between POFS and PSFS and work engagement, the association between work engagement and subjective well-being, and the mediating role of work engagement between POFS and PSFS and subjective well-being among employees working remotely and on-site during the COVID-19 pandemic.

The SEM analysis revealed a significant positive correlation between POFS and work engagement for teleworking employees and those working on-site. These findings are supported by scholars such as Aryee et al. [54] and Wayne et al. [64]. The primary rationale behind this is that POFS serves as a resource that facilitates the acquisition of additional resources, offering professionals the flexibility to manage both work and family demands. Within the motivational framework of the Job Demands Resources (JD-R) theory, the support extended by organisations to employees’ non-work domains promotes an increase in another resource, such as work engagement. Moreover, as noted by Demerouti and Bakker [55], in a crisis such as the COVID-19 pandemic, other resources beyond job characteristics may assume a more relevant role. Thus, organisational family support may assume higher importance in explaining work engagement than in non-crisis situations. Future studies should explore this relationship in a non-crisis situation to determine whether the same pattern of results can be obtained.

Furthermore, the study identified a significant positive relationship between PSFS and work engagement among telecommuting employees. Consistent with prior research, the results obtained from the teleworker sample align with scholars’ assertions regarding the relationship between PSFS and work engagement [54,86]. Thus, the presence of resources such as PSFS will foster the accumulation of other resources, leading to more positive results, such as higher work engagement [67]. However, contrary to our expectations, a similar relationship pattern was not observed among on-site employees. This outcome could be attributed to the unprecedented circumstances faced by on-site workers during the COVID-19 pandemic. Specifically, on-site workers experienced heightened concerns about contagion and had to adapt to new social interaction norms during this period [36]. Consequently, the support offered by supervisors aimed at safeguarding employees’ health while at work may have assumed greater significance in enhancing work engagement compared to supervisory family support. Thus, it is essential to replicate this study in a non-crisis situation. 

The findings from the current study supported the hypothesis that work engagement would exhibit a positive relationship with subjective well-being for teleworkers and on-site employees. These observations align with findings from previous studies, such as those by Ouweneel et al. [102] and Tokdemir [103]. Therefore, experiencing a positive state at work, such as work engagement, contributes to positive outcomes beyond the workplace, ultimately enhancing individuals’ overall well-being, as outlined by Robertson and Cooper [101].

Concerning the mediating role of work engagement in explaining the relationship between sources of support (i.e., POFS and PSFS) and subjective well-being, the current research broadly highlights the mediating role of work engagement for teleworkers and on-site workers. The only exception was verified in the on-site workers concerning the mediating role of work engagement in explaining the relationship between PSFS and subjective well-being, where this mediating role was not observed. As previously mentioned, this outcome may be attributed to the exceptional circumstances encountered by on-site workers amid the COVID-19 pandemic. Throughout this period, on-site workers had to face the pandemic within their workplace environments, adhering to social distancing guidelines and implementing enhanced personal hygiene and sanitation practices, which heightened the job demands for on-site workers [74]. Given these circumstances, PSFS may have assumed less relevance in explaining work engagement and subjective well-being since the support provided by supervisors towards dealing with the workplace environment during the COVID-19 pandemic may have assumed greater importance for on-site workers. Therefore, the mediating role of work engagement in explaining the relationship between PSFS and subjective well-being was not observed for on-site workers. 

When considering the limitations, it is essential to consider certain aspects for future research. Firstly, this study was conducted amidst the COVID-19 pandemic. Although the findings yielded insights into the relationship patterns amid a crisis, wherein the significance of support sources linked to non-work domains (i.e., POFS and PSFS) is notably accentuated, it is paramount to replicate this study in a non-crisis context to ascertain whether analogous relationship patterns arise. Secondly, the sample consisted solely of employees from Portugal, potentially limiting the generalisability of the findings. Hence, it is recommended that the geographical scope of the study be expanded to encompass non-European countries. In addition, the different sizes of the two subgroups—teleworkers and on-site workers—in the current study may also have limited the findings. In the future, it is essential to replicate this study with a more balanced sample size between the two groups and in a context without a crisis. Another aspect to note is the cross-sectional design of this study, precluding the establishment of causality. Future studies employing longitudinal designs are encouraged to surmount this limitation. Furthermore, each variable was solely evaluated using self-report measures, raising concerns regarding common method bias. Nonetheless, given that all variables pertained to individuals’ perceptions and were centred on personal experiences, self-report measures appeared to align better with the primary research objectives. However, concerning the assessment of individuals’ well-being, it would be beneficial to incorporate objective indicators, such as health metrics (e.g., blood pressure), in future investigations.

## 5. Conclusions

This study has provided valuable insights into the intricate relationships between perceived organisational family support (POFS), perceived supervisory family support (PSFS), work engagement, and subjective well-being among employees amidst the challenging backdrop of the COVID-19 pandemic.

The present study’s findings reveal a significant positive correlation between POFS and work engagement among teleworking employees and those working on-site. These results are consistent with previous research conducted by Aryee et al. [54] and Wayne et al. [64], indicating that POFS plays a pivotal role as a resource in augmenting worker engagement within the framework of the Job Demands Resources (JD-R) theory.

Furthermore, the current study identified a significant positive relationship between PSFS and work engagement among telecommuting employees, consistent with the suggestions in the works of Aryee et al. [54] and Greenhaus et al. [86]. However, contrary to our expectations, a similar relationship pattern was not observed among on-site employees, possibly due to the unprecedented circumstances faced by on-site workers during the pandemic, including heightened concerns about contagion and adjustments to new social interaction norms.

Regarding subjective well-being, our findings support the hypothesis that work engagement is positively associated with subjective well-being for teleworkers and on-site employees, aligning with previous studies by Ouweneel et al. [102] and Tokdemir [103].

Furthermore, our research underscores the mediating role of work engagement in explaining the relationship between POFS and subjective well-being for teleworkers and on-site workers. However, this mediating role was not observed among on-site workers concerning the relationship between PSFS and subjective well-being, potentially attributed to the heightened job demands and workplace challenges that on-site workers faced during the pandemic.

Considering these findings, future research must replicate this study in a non-crisis context and expand the geographical scope beyond Portugal to enhance the generalisability. Longitudinal studies are recommended to establish causality, and incorporating objective indicators, such as health metrics, alongside self-report measures would provide a comprehensive understanding of individuals’ well-being.

Overall, this study contributes valuable insights into the dynamics of organisational and supervisory support, work engagement, and subjective well-being, offering implications for theory, practice, and future research in organisational psychology and employee well-being.

## Figures and Tables

**Table 1:** Demographics of the sample.

Variables	Total Sample (*n* = 515)	Teleworkers’ Sample (*n* = 411)	On-Site Workers (*n* = 104)
Women (%)	74.6	76.6	66.3
Age (Mean)	39.87 (SD = 9.45)	39.56 (SD = 9.08)	41.08 (SD = 10.72)
Education			
9th grade (%)	2.7	1.7	6.7
12th grade	19.4	16.3	31.7
Bachelor’s degree (%)	48.5	51.3	37.5
Master’s degree (%)	26.6	27.7	22.1
Doctoral degree (%)	2.7	2.9	1.9
Civil status			
Single (%)	29.4	29.9	27.5
Married (%)	70.6	70.1	72.5
Have children (% Yes)	62.7	62.3	64.4
Permanent employment contract (%)	74.0	76.9	62.5
Job tenure			
Less than 1 year (%)	15.5	15.1	17.3
Between 2 and 3 years (%)	21.6	22.6	17.3
Between 4 and 5 years (%)	12.8	12.9	12.5
Between 6 and 10 years (%)	13.2	13.4	12.5
More than 10 years (%)	36.9	36.0	40.4

**Table 2:** Mean comparison between groups.

Variables	Teleworkers (*n* = 411)		On-Site Workers (*n* = 104)		Test to Compare Groups
	Mean	*SD*	Mean	*SD*	
POFS	4.40	1.51	3.66	1.56	*t* = 4.39, *p* < 0.01
PSFS	5.73	1.10	5.20	1.22	*t* = 4.33, *p* < 0.01
Work Engagement	5.25	1.01	5.07	1.12	*t* = 1.58, n.s.
OLS	7.88	1.35	7.63	1.57	*t* = 1.66, n.s.
PWI	7.75	1.18	7.55	1.13	*t* = 1.58, n.s.

Note: POFS = perceived organisational family support; PSFS = perceived supervisory family support; PWI = Personal Well-being Index; OLS = Overall Life Satisfaction; SD = standard deviation.

**Table 3:** Correlation matrix.

	1.	2.	3.	4.	5.	6.	7.	8.	9.	10.	11.
*r* for teleworkers (below the diagonal) and for on-site workers (above the diagonal)											
1. Sex		−0.12	0.25 *	0.05	−0.06	−0.15	−0.18	−0.15	−0.15	−0.01	0.01
2. Age	−0.03		−0.39 **	−0.40 **	0.64 **	0.64 **	−0.04	0.01	0.29 **	−0.06	−0.12
3. Education	0.04	−0.25 **		0.03	−0.24 *	−0.23 *	−0.05	−0.06	−0.02	−0.02	0.07
4. Single	−0.03	−0.40 **	0.19 **		−0.56 **	−0.18	0.01	−0.03	0.01	0.01	−0.03
5. Children	0.13 **	0.51 **	−0.18 **	−0.66 **		0.39 **	−0.04	0.08	0.07	0.05	−0.11
6. Tenure	−0.06	0.58 **	−0.26 **	−0.30 **	0.36 **		−0.11	−0.14	0.16	−0.09	−0.05
7. POFS	−0.13 *	0.04	−0.10 *	−0.04	−0.01	−0.05		0.56 **	0.40 **	0.26 **	0.23 *
8. PSFS	−0.06	−0.08	−0.05	0.02	−0.05	−0.07	0.58 **		0.21 *	0.22 *	0.08
9. Work Engagement	−0.12 *	0.17 **	−0.02	−0.09	0.06	0.11 *	0.34 **	0.32 **		0.34 **	0.35 **
10. OLS	0.05	−0.03	0.07	−0.06	0.00	0.01	0.24 **	0.23 **	0.38 **		0.66 **
11. PWI	0.03	−0.11 *	0.07	0.06	−0.13 *	−0.05	0.28 **	0.23 **	0.36 **	0.74 **	

Note: POFS = perceived organisational family support; PSFS = perceived supervisory family support; PWI = Personal Well-being Index; OLS = Overall Life Satisfaction; dummy variables: sex (0 = men; 1 = women), single (1 = yes; 0 = no), and children (1 = yes; 0 = no). ** *p* < 0.01; * *p* < 0.05.

**Table 4:** Indirect effects for mediation models.

	Indirect Effects			
	Estimates (SE)		Bias-Corrected 95% CI	
	Teleworkers (n = 411)	On-Site Workers (n = 104)	Teleworkers (n = 411)	On-Site Workers (n = 104)
POFS → Work Engagement → Subjective Well-Being (PWI measure)	0.07 (0.03)	0.15 (0.06)	0.03; 0.14	0.05; 0.33
POFS → Work Engagement → Subjective Well-Being (OLS measure)	0.09 (0.03)	0.12 (0.07)	0.04; 0.16	0.01; 0.31
PSFS → Work Engagement → Subjective Well-Being (PWI measure)	0.07 (0.03)	−0.03 (0.04)	0.02; 0.14	−0.13; 0.03
PSFS → Work Engagement → Subjective Well-Being (OLS measure)	0.09 (0.03)	−0.02 (0.04)	0.03; 0.16	−0.13; 0.02

Note: POFS = perceived organisational family support; PSFS = perceived supervisory family support; PWI = Personal Well-being Index; OLS = Overall Life Satisfaction; CI = confidence interval. The standard error (SE) is shown in parentheses. We used 5000 bootstrap samples with a 95% bias-corrected bootstrap confidence interval for all indirect effects.

## Data Availability

The data that support the findings of this study are available from the corresponding authors, S.L. and V.S.C., upon reasonable request.
